# Effects of Different Prebiotics on the Gel Properties of Milk Protein and the Structural Features of Yogurt

**DOI:** 10.3390/gels9110863

**Published:** 2023-10-30

**Authors:** Dongdong Li, Mengxuan Lai, Pengjie Wang, Hairan Ma, Hongliang Li, Ran Wang, Xiuying Wu

**Affiliations:** 1 Mengniu Hi-Tech Dairy Product Beijing Co., Ltd., Beijing 101100, China; lidongdong0826@163.com; 2 Beijing Advanced Innovation Center for Food Nutrition and Human Health, Department of Nutrition and Health, China Agricultural University, Beijing 100190, China; wpj1019@cau.edu.cn; 3 Inner Mongolia Mengniu Dairy (Group) Co., Ltd., Hohhot 011500, China; laimengxuan@mengniu.cn (M.L.); mahairan@mengniu.cn (H.M.); lihongliang@mengniu.cn (H.L.)

**Keywords:** prebiotics, yogurt, rheology, texture, gel properties

## Abstract

The impact of prebiotics on the structural characteristics of yogurt is an important aspect of evaluating its functional properties. This study aimed to evaluate and compare the effects of several commonly used prebiotics, including fructooligosaccharide (FOS), galactooligosaccharide (GOS), inulin (INU), polydextrose (PDX), and xylooligosaccharide (XOS), on the gel properties of milk protein and the structural features, with respect to the texture and rheology, of stirred yogurt during and after fermentation. The results revealed that the supplementation of INU, PDX, and XOS was involved in the construction of protein networks during fermentation, promoting a viscous and more elastic gel structure, due to the enhanced protein–water interactions. This resulted in a significant increase (*p* < 0.05) in structural stability (higher critical strain (γ_c_) and greater thixotropy), firmness, cohesiveness, and rheology (G′ and G″) and a significant decrease (*p* < 0.05) in the loss of yogurt during centrifugation. Conversely, the supplementation of GOS and FOS did not appear to be involved in the construction of the protein network and barely affected the rheological properties of the gel during fermentation. However, a significant increase (*p* < 0.05) in viscosity and firmness, and a slight decrease (*p* > 0.05) in loss during centrifugation were still observed in the yogurt. These findings could be useful for a comprehensive assessment of the application potential of these prebiotics in yogurt, when combined with their respective prebiotic properties.

## 1. Introduction

Yogurt is considered a healthy and nutritious food due to its good sensory properties, rich bioactive substances (hydrolyzed proteins, carbohydrates, vitamins, and minerals), and high bioavailability [1]. It is a dairy product fermented by a starter culture consisting of *Lactobacillus delbrueckii* ssp. *bulgaricus* (LB) and *Streptococcus thermophilus* (ST) [2]. During fermentation, milk protein is hydrolyzed by the proteolytic enzymes produced by the starter culture bacteria, and lactose is converted into lactic acid by the lactic acid bacteria. This acidifies the pH of the milk from about 6.5 to about 4.5, causing caseins to aggregate and form a three-dimensional network, endowing yogurt with a unique texture and rheological properties [2].

In recent years, with the upgrading of healthy consumption concepts, the development of prebiotic fortified yogurt products has become increasingly popular [3]. On the one hand, this is attributed to the maintenance effect of prebiotics on a healthy gut microbiota or the reconstitution/balancing effect on a destabilized gut microbiota [4]. On the other hand, this is also related to the substitution of prebiotics for fat [5] or sugar (as a low-calorie sweetener) [6] in yogurt products, as well as their regulatory effects on texture and rheology [3,6]. Fructooligosaccharide (FOS), galactooligosaccharide (GOS), inulin (INU), polydextrose (PDX), and xylooligosaccharide (XOS) are common types of prebiotics [6,7], which can provide various functional properties when incorporated into food systems [6]. The textural and rheological properties of yogurt are considered to be important attributes affecting consumer acceptability [2], which are influenced by the functional properties of prebiotics. These functional properties involve water retaining, gel-forming, fat mimetic, anti-sticking, anti-clumping, texturizing, and thickening, and ultimately contribute to changes in the structural characteristics of yogurt products [7]. The concentration, category, and interaction of prebiotics with other food ingredients are considered to be key factors influencing the type and extent of prebiotic functional effects [6,7]. Cruz et al. [8] reported that an increasing concentration (2–8%, *wt*/*v*) of oligofructose resulted in an increase in the thixotropy, apparent viscosity, storage modulus, and loss modulus of yogurt under initial storage conditions (5 °C). However, when its concentration level exceeded 4%, it could impede the formation of a protein network, showing weak gel behavior and reduced gel strength. Likewise, Paseephol et al. [9] suggested that yogurt with 4% added inulin showed a decrease in gel strength. In addition, Guggisberg et al. [5] found that the yield stress, sensory firmness, and creaminess of set yogurt with different fat levels (1–3.5%) increased with rising levels of inulin addition (0–4%), and that the largest yield stress was observed for 4% inulin addition. Similarly, Srisuvor et al. [10] indicated that increasing the concentration of polydextrose (1–3%, *wt*/*v*) improved the physical (syneresis, texture, and apparent viscosity) and sensory properties of low-fat set yogurt to varying degrees, with 2 g polydextrose/100 mL being the most appropriate level. According to previous research [2,5,7,10,11], these commonly used prebiotics in yogurt formulas, including FOS, GOS, INU, PDX, and XOS, were typically added in amounts ranging from 2% to 5% (*wt*/*v*) to improve the flavor, texture, rheology, and growth of probiotics in yogurt, and 2.5% (*wt*/*v*) was commonly considered an appropriate level of prebiotics added as a functional strengthening component in yogurt. Additionally, prebiotics could also affect the fermentation process through the water–protein interaction induced by the hygroscopic ability of prebiotics [6], thereby affecting the development of the textural and rheological properties of yogurt products. Therefore, it is also necessary to compare and evaluate the impacts of different prebiotics on the structural characteristics of yogurt itself, before they are selected as functional components for supplementation, in addition to considering their respective prebiotic functional effects.

In this study, the impacts of several common prebiotics (FOS, GOS, INU, PDX, and XOS) on the textural and rheological properties of stirred yogurt during and after fermentation at the same dosage (2.5 g/100 mL) were investigated. The main purpose was to provide important complementary information on the structural properties of yogurt with several commonly used prebiotics.

## 2. Results and Discussion

### 2.1. Rheological Characteristics

#### 2.1.1. Microrheological Properties

Casein gel is the main component of yogurt structure and has dynamic properties [12]. The excessive rearrangement of gel particles making up the gel network, before and during gelation, is responsible for the fermentation process [13]. MS-DWS is a microrheological technique that is often used to study the gelation mechanism of yogurt during fermentation [14,15,16]. In this study, three parameters derived from MS-DWS, elasticity index (EI), macroscopic viscosity index (MVI), and solid liquid balance (SLB), were used to monitor the gelation process of the control and prebiotic yogurts during fermentation for about 4 h. The EI is directly proportional to the elastic modulus as a function of time, and the MVI is directly proportional to the viscosity modulus as a function of time [17].

**Figure 1:**
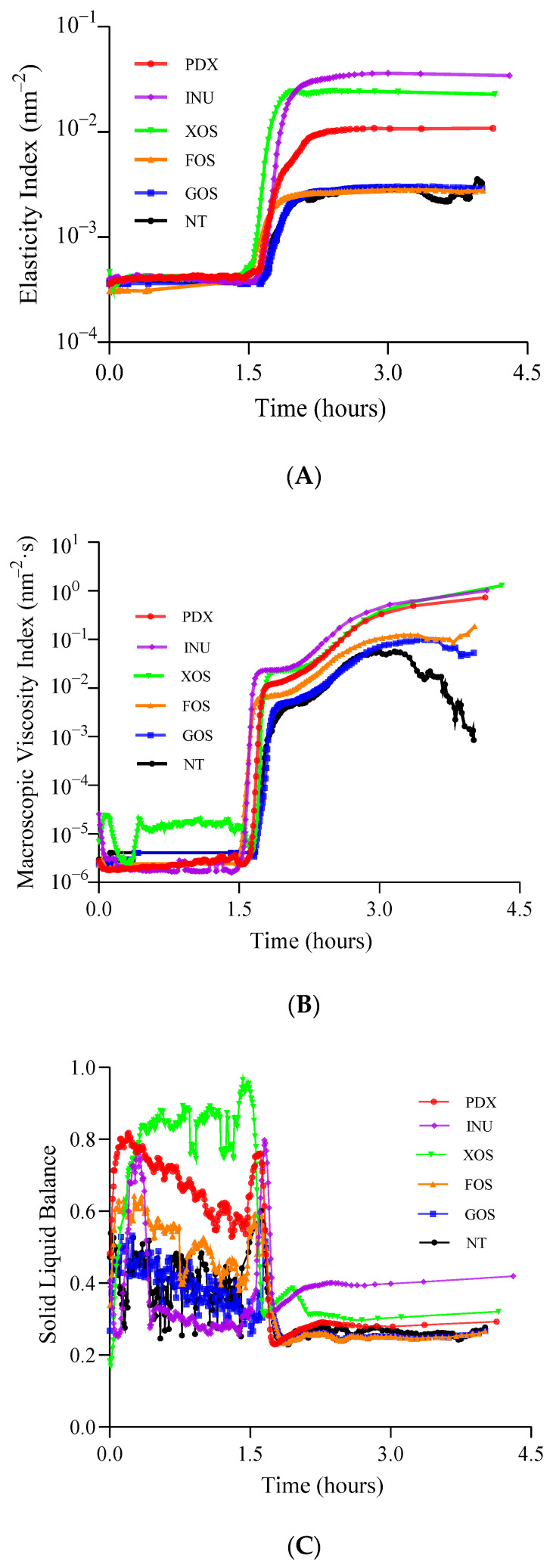
Microrheological properties of control and probiotic yogurts during fermentation. Elastic index (**A**), macroscopic viscosity index (**B**), and solid–liquid balance (**C**); NT = control, GOS = galactooligosaccharide, FOS = fructooligosaccharide, XOS = xylooligosaccharide, INU = inulin, PDX = polydextrose.

As shown in Figure 1A,B, the EI and MVI values of the yogurt samples in each group remained basically stable at about 0–1.5 h, except for a small downward peak in the XOS-added group. At this stage, casein had not yet formed a gel structure, but it began to dissociate with microbial acidification [12]. As the fermentation time extended, the EI and MVI rapidly increased to their maximum values at about 1.5–2.0 h due to successively reaching the gel point (pH = 4.60), and then remained stable (EI) or slightly increased (MVI) at about 2.0–4.0 h, except for the MVI of the control sample. At this stage, with the increase in acid production by the starter bacteria, casein gradually approached its isoelectric point, the repulsions between casein particles decreased, the hydrophobic interactions strengthened, and then casein began to aggregate and form a gel structure [18]. In particular, the initial gel times of the yogurt groups varied from about 1.5 h to 2.0 h, which may be due to the different effects of prebiotics on the acid kinetics of the starter bacteria [4]. Heydari et al. [19] reported that the addition of prebiotics/fibers had different effects on the acidification rate and pH decrease during fermentation, depending on the type and level of prebiotics added. The SLB provides an indication of the ratio between the solid-like and liquid-like behavior of yogurt as a function of time and is directly proportional to the viscoelasticity of the sample [17]. As shown in Figure 1C, the SLB of all yogurt groups fluctuated greatly initially at about 0–1.5 h, then dropped sharply to the minimum (<0.5 in all cases) at about 1.5–2.0 h, and remained basically stable at about 2.0–4.0 h. This suggested that the formed gels of all yogurt groups could be predominantly elastic- or solid-like, according to the indications that the yogurts were solid-like and liquid-like when the SLB was 0–0.5 and 0.5–1, respectively, as reported by He et al. [13]. On the other hand, during and after gel formation (about at 1.5–4.0 h), the EI and MVI values were similar between the GOS-added, FOS-added, and control yogurt groups, but they were all lower than those of the INU-added, XOS-added, and PDX-added yogurt groups. Gomes et al. [20] reported that soluble dietary fibers can be adsorbed on casein micelles and further integrated into the structure of yogurt, participating in the stabilization of the protein network structure during acidification. Therefore, the samples with added INU and PDX could form a relatively denser structure due to their interactions with milk protein components [7], endowing the yogurt with higher gel structural strength [11]. These interactions can be non-covalent, with an attractive or repulsive nature, including electrostatic, hydrophobic, hydrogen, and Van der Waals forces [20]. In addition, the stable yogurt structure and the hygroscopicity of the prebiotics themselves also prevent the migration of water from the protein network, resulting in a more viscous and rigid gel property [20], as indicated by their higher MVI and EI values.

In general, the hygroscopicity and structural strengthening brought by prebiotics to yogurt affected the rheological properties before gelation and the rigidity and viscosity of the network structure after gelation, to a certain extent. These effects could further contribute to the development of the rheological properties of stirred yogurt, as the degree of cohesion and compaction of the original gel structure would influence the viscoelasticity and structural stability of yogurt after stirring, and these properties could be further characterized by frequency sweep and amplitude sweep.

#### 2.1.2. Frequency Sweep

A viscoelastic behavior was observed in the yogurt samples through the oscillatory test, which can be determined by the measurement of the elastic modulus (G′) and viscous modulus (G″). G′ is defined as a measure of the energy stored per oscillation cycle and can be used as an indicator of the stiffness or elasticity of the material [6]. G″ is a measure of the energy dissipated or lost per oscillation cycle. The relative sizes of both determine the viscoelastic characteristics of the sample [6]. If G′ is greater than G″, the sample will behave more like a solid, meaning that deformation will be essentially elastic or recoverable. However, if G″ is greater than G′, the energy used to deform the sample is dissipated viscously and the sample will behave like a liquid [11]. The rheological behavior of yogurt is the result of the three-dimensional gel network formed by casein and denatured whey proteins [21]. During fermentation, acidification leads to the formation of protein aggregates through hydrophobic interactions and electrostatic bonds, which are further cross-linked to form a gel structure [6]. Total solids and protein content can affect the gel strength of the final yogurt product [22]. The addition of prebiotics increased the dry matter content of yogurt [18], and affected the gel structural properties and rheological behavior of the product [23].

**Figure 2:**
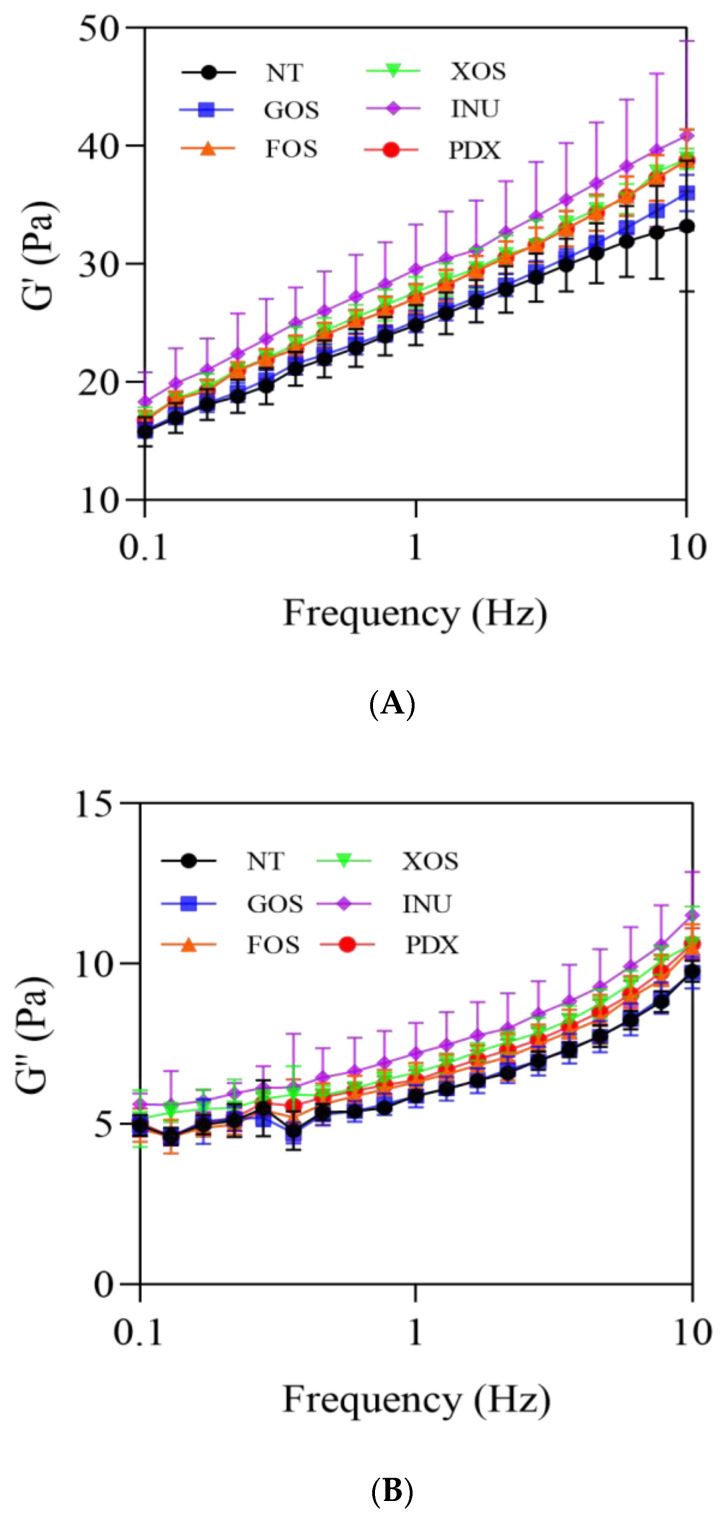
Frequency sweep curves of control and probiotic yogurts. (**A**) Storage modulus G′; (**B**), loss modulus G″. NT = control, GOS = galactooligosaccharide, FOS = fructooligosaccharide, XOS = xylooligosaccharide, INU = inulin, PDX = polydextrose.

As shown in Figure 2A,B, the G′ and G″ for the yogurt samples are frequency-dependent, and gradually increased with an increasing frequency. All yogurt samples exhibited viscoelastic properties, with G′ being higher than G″ across the frequency range. However, no pronounced differences in magnitude or trend with respect to G′ or G″ were observed between the different prebiotic yogurt groups and the control group across the frequency range. Nevertheless, the highest G′ and G″ levels were observed in the INU-added yogurt group, intermediate G′ and G″ levels were observed in the XOS-added and PDX-added yogurt groups, and the lowest G′ and G″ levels were in the GOS-added and FOS-added yogurt groups. An increase in G′ and G″ indicated a more strongly structured protein network with more bonds [8] or a gel structure with increased intermolecular cross-linkings [11]. FOS, GOS, INU, PDX, and XOS are commonly used soluble dietary fibers, and they have different water retention capabilities due to differences in structure and composition [7]. Samakradhamrongthai et al. [24] revealed that, when the soluble fiber binds to water molecules, these water molecules also interact with milk proteins to result in a more viscous gel with more and stronger intermolecular interactions. In the present study, the PDX-added, XOS-added, and INU-added yogurt samples had higher water retention than the control, GOS-added, and FOS-added samples (as indicated in Section 2.3), among which inulin (INU) had the most prominent ability, probably because its structure is composed of a microcrystalline network formed of small aggregates, which are capable of retaining water [25].

#### 2.1.3. Amplitude Sweep

**Figure 3:**
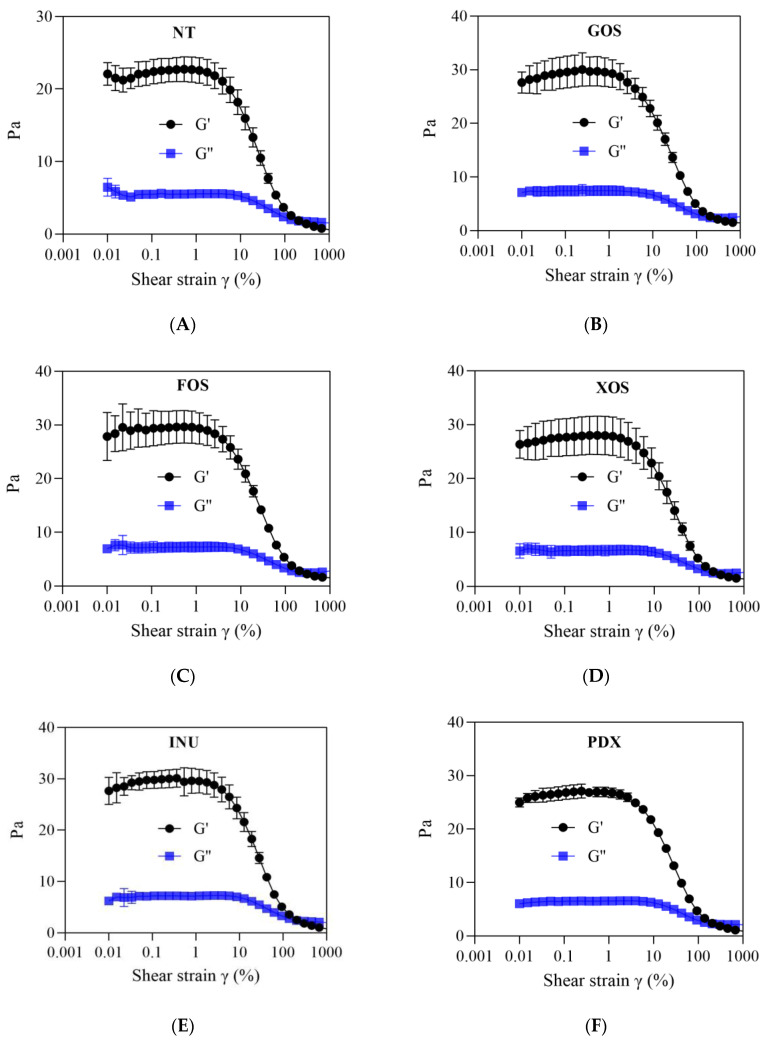
Amplitude sweep curves of control and probiotic yogurts. NT = control (**A**), GOS = galactooligosaccharide (**B**), FOS = fructooligosaccharide (**C**), XOS = xylooligosaccharide (**D**), INU = inulin (**E**), PDX = polydextrose (**F**).

As shown in Figure 3, the variations in G′ and G″ of the control and prebiotic yogurts as a function of increasing strain are provided by amplitude sweep. The maximum strain value that the sample can withstand without affecting its own structure can be obtained through amplitude sweep, which is defined as the critical strain (γ_c_) [26].

As shown in Figure 3A–F, in the critical strain region, the G′ and G″ measures of each group of yogurt samples did not change significantly with the increase in strain, and G′ was always greater than G″. This indicated that the gel network structure of the samples was not destroyed in this strain range and exhibited relatively strong elastic or solid properties. When further increasing the strain to reach or exceed the critical strain (γ_c_) of the samples, both G′ and G″ began to decrease gradually until they intersected and presented a G′–G″ crossover point. The values of G_c_′ and G_c_″ at critical strain and the γ_c_ values of all yogurt groups are listed in Table 1.

We observed that the γ_c_ values of the INU-added, XOS-added, and PDX-added yogurt groups were higher than those of the GOS-added (*p* < 0.05), FOS-added (*p* < 0.05), and control yogurt groups (*p* > 0.05). Moreover, the G_c_′ and G_c_″ levels of the prebiotic yogurt groups were also higher than those of the control group (*p* < 0.05), except for the PDX-added yogurt group (*p* > 0.05). The increase in γ_c_, G_c_′, and G_c_″ in the samples reflects the enhanced stability of their gel structures [27]. Guénard-Lampron et al. [27] indicated that the addition of polydextrose and inulin promoted more intermolecular interactions between the constituents of yogurt products. Costa et al. [7] proposed that yogurts containing inulin and polydextrose presented higher interaction factors, including intermolecular interactions (hydrogen bonds), hydrophobic interactions (between casein molecules and fat), and electrostatic calcium bridges. This led to greater gel strength and higher gel stability of these prebiotic yogurt groups.

The contribution of the interaction of prebiotic and protein constituents in yogurt to the connections between gel particles can also be reflected as the resistance of the gel structure to shear. More intramolecular connections and stronger interactions endowed yogurt gels with higher structural stability and less fluidity, which in turn exhibited higher shear resistance and viscous properties at the macroscopic level, and these characteristics were further characterized through changes in thixotropy and viscosity.

### 2.2. Thixotropic and Viscous Properties

**Figure 4:**
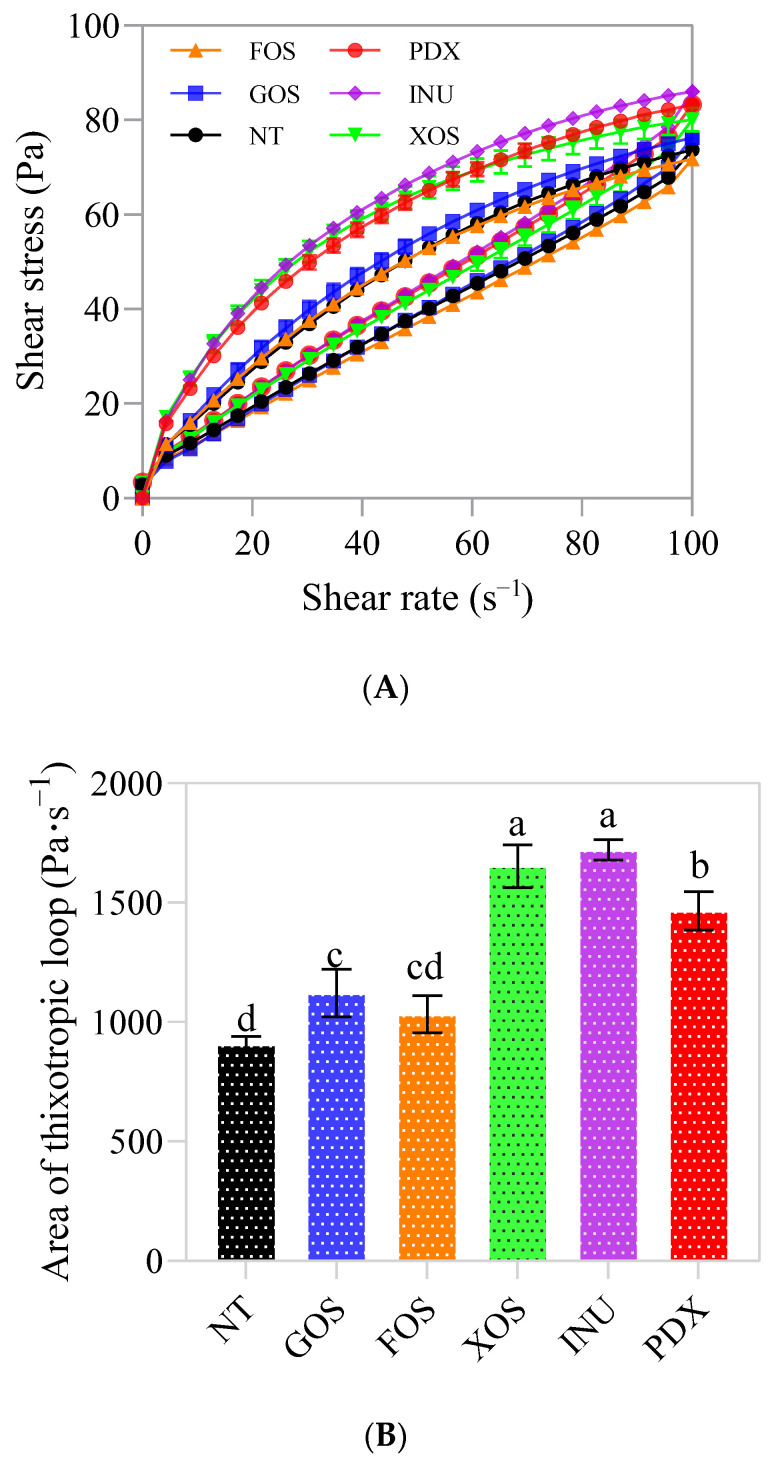
The thixotropic properties (**A**) and thixotropic loop area (**B**) of control and probiotic yogurts. NT = control, GOS = galactooligosaccharide, FOS = fructooligosaccharide, XOS = xylooligosaccharide, INU = inulin, PDX = polydextrose. Different letters in (**B**) represent significant differences (*p* < 0.05).

Thixotropy can be detected in the fragile structure of a typical non-Newtonian fluid, such as yogurt, when its three-dimensional gel network is destroyed by shear [8]. As shown in Figure 4A, when the control and prebiotic yogurt samples were sheared at a rate that first increased and then decreased, the shear stress first increased to a maximum value and then decreased. The yogurt samples exhibited typical shear-thinning and thixotropic behavior [28], i.e., irreversible structural changes over time under shear. The area enclosed by the upward curve and the downward curve is called the thixotropic loop [8]. The formation of thixotropic loops can reflect changes in the rheological behavior of yogurt. Also, the energy required to break the structure of yogurt is proportional to the area of the thixotropic loops [11]. As shown in Figure 4B, the areas of thixotropic loops in different prebiotic yogurt groups were higher than those in the control group, among which the highest area values were obtained by the INU-added (*p* < 0.05) and XOS-added (*p* < 0.05) yogurt groups, followed by the PDX-added yogurt group (*p* < 0.05), and finally by the GOS-added (*p* < 0.05) and FOS-added (*p* > 0.05) yogurt groups, when compared to the control group. Similarly, Debon et al. [11] and Cruz et al. [8] reported that dairy products containing inulin and oligofructose exhibited higher thixotropic loop areas than the control group. Costa et al. [7] reported that yogurt with added inulin, xylooligosaccharide, and polydextrose had higher shear stress values than traditional yogurt and yogurt with added fructooligosaccharide. The increase in thixotropy of the yogurt samples with added prebiotics was considered to be possibly related to the increase in viscosity. As stated by Costa et al. [7] and Vargas et al. [29], a high-viscosity thixotropic fluid, necessarily, usually has a larger hysteretic area than a low-viscosity thixotropic fluid.

**Figure 5:**
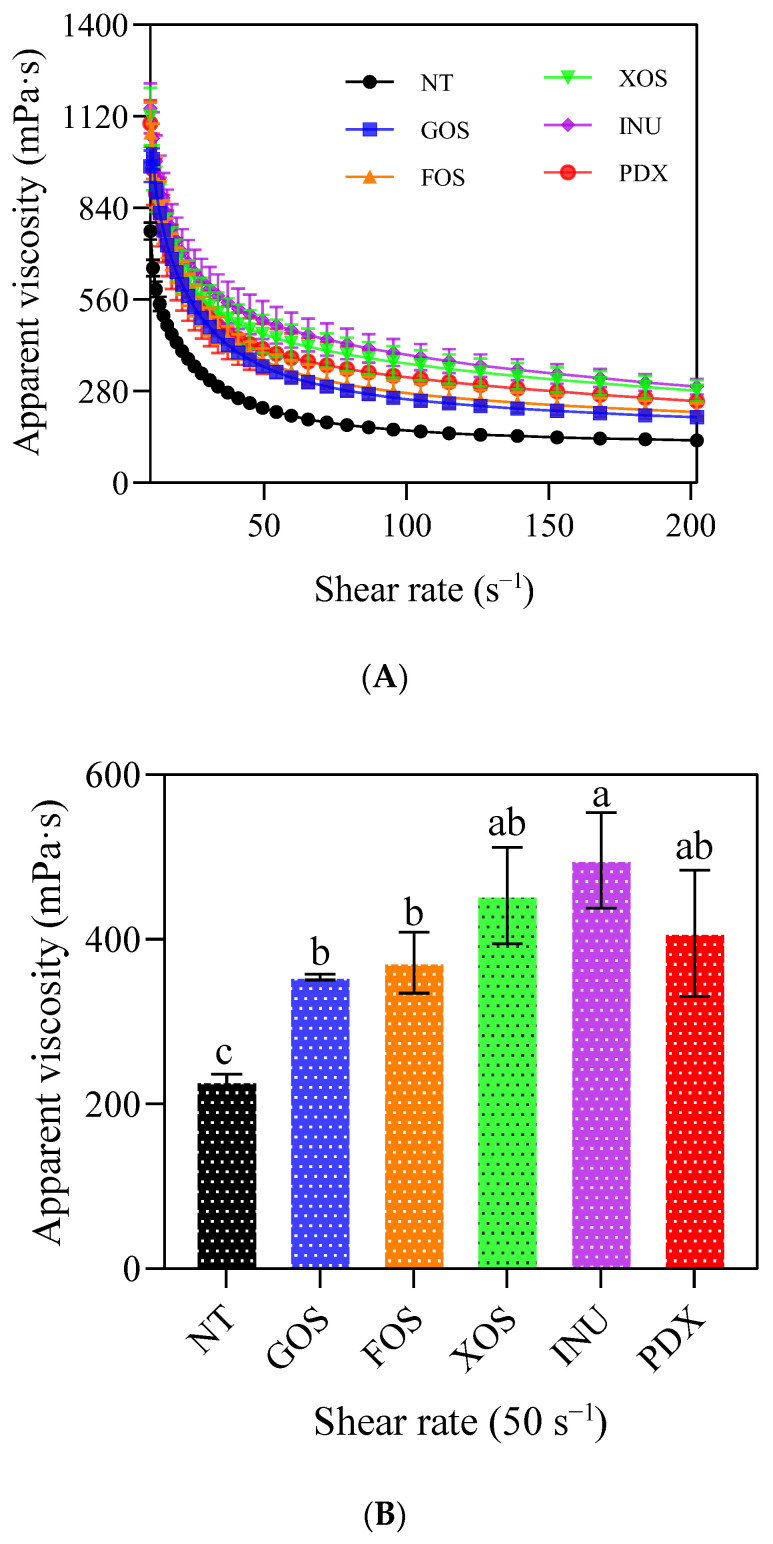
Changes in apparent viscosity of control and probiotic yogurts at 0–200 s^−1^ (**A**) and 50 s^−1^ (**B**). NT = control, GOS = galactooligosaccharide, FOS = fructooligosaccharide, XOS = xylooligosaccharide, INU = inulin, PDX = polydextrose. Different letters in (**B**) represent significant differences (*p* < 0.05).

As shown in Figure 5A, the different prebiotic yogurt groups had higher initial viscosity than the control yogurt, and it decreased sharply with the increase in shear rate, exhibiting a typical pseudoplastic behavior [30]. This behavior may be due to the fact that macromolecules such as proteins and fats in yogurt tend to orient themselves with the movement of the fluid, thus reducing flow resistance with an increase in shear strain rate [6]. The addition of different prebiotics had different effects on the flow behavior of yogurt. Balthazar et al. [6] reported that adding inulin and fructo-oligosaccharide to sheep milk ice cream prominently decreased the flow behavior index and increased the consistency of the samples compared to the control group. However, sheep milk ice cream with added galacto-oligosaccharide and short chain fructo-oligosaccharide and polydextrose showed better fluidity (tan σ > 1), possibly due to a less structured protein network with fewer bonds, or increased mobility of the molecules [6].

Viscosity at a shear rate of 50 s^−1^ (η_50_) is widely considered to correlate well with perceived thickness, stickiness, and sliminess of a variety of food products, from Newtonian fluids to thick emulsions [31]. As can be seen from Figure 5B, the apparent viscosity at 50 s^−1^ of each prebiotic yogurt group was significantly higher than that of the control group (*p* < 0.05). Among them, the INU-added yogurt group showed the highest values of η_50_, followed by the XOS-added and PDX-added yogurt groups, and finally by the GOS-added (*p* < 0.05) and FOS-added (*p* > 0.05) yogurt groups. Balthazar et al. [6] reported that an increase in viscosity and consistency was to be expected in the formulation of sheep milk ice cream containing inulin, because inulin is highly hygroscopic, which leads to strong water–protein interactions, forming a more viscous gel-like network. In another published study [7], the rheological properties of several Greek yogurts supplemented with different prebiotics were evaluated from the perspective of differences in particle size distribution of these different prebiotics themselves. More viscous and less fluid features were observed in the Greek yogurts with added galactooligosaccharide, inulin, and polydextrose, due to the larger particle size of these prebiotic components; an increased flow behavior index was found in the Greek yogurt with added fructooligosaccharide, due to the lubrication effect generated by the small particle size of this prebiotic; no effect was observed in the Greek yogurt with added xylooligosaccharide, due to the intermediate particle size of this prebiotic. These findings may indicate that the thixotropy, viscosity, and flow behavior of yogurt share a common structural basis: that is, the breakdown of weak bonds and the reduction in the sum of electrostatic repulsion and hydrophobic interactions between gel molecules [11], due to the shear force.

### 2.3. Texture and Water-Retaining Properties

Texture is a group of physical properties that depend on the structure of food and the way its components interact [5].

**Figure 6:**
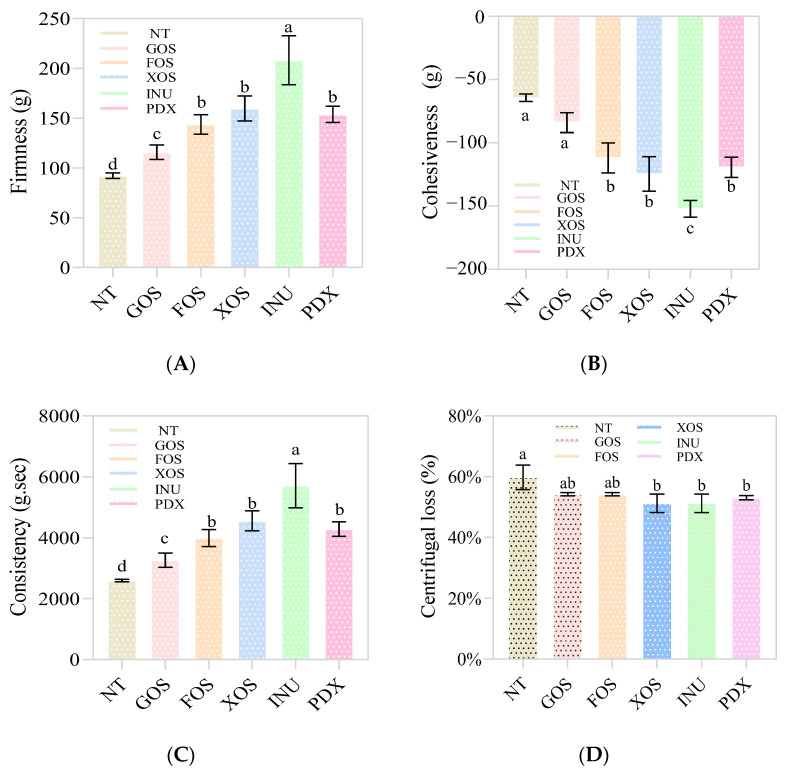
Texture and centrifugation loss of control and probiotic yogurt. NT = control, GOS = galactooligosaccharide, FOS = fructooligosaccharide, XOS = xylooligosaccharide, INU = inulin, PDX = polydextrose. (**A**–**D**) represents the change in firmness (**A**), cohesiveness (**B**), consistency (**C**) and centrifugal loss (**D**), respectively. Different letters represent significant differences (*p* < 0.05).

As depicted in Figure 6A–C, the texture parameters of the different yogurt groups, including firmness, consistency, and cohesiveness, showed a similar trend: that is, the texture parameters of the prebiotic yogurt groups were significantly higher than those of the control group (*p* < 0.05). Among them, the highest parameter levels were observed in the INU-added yogurt group, followed by the XOS-added, PDX-added, and FOS-added yogurt groups, and finally the GOS-added yogurt group. Dias et al. [32] reported that the addition of polydextrose resulted in Greek yogurt with greater cohesiveness by promoting a greater number of interactions between product ingredients. Balthazar et al. [6] reported that sheep milk ice cream samples, to which inulin and fructooligosaccharide were added, exhibited higher complex modulus and firmness due to increased gel strength. Likewise, Costa et al. [7] reported that the addition of inulin, polydextrose, and galactooligosaccharide resulted in higher gel strength, consistency, and firmness of Greek yogurt, when compared with the traditional formula without prebiotics. This prebiotic-induced increase in the textural properties of yogurt was more attributed to the increase in intermolecular interactions [7] such as hydrogen bonds, hydrophobic interactions, and electrostatic calcium bridges between casein, which provided greater gel strength and consequent greater rigidity and consistency [6]. Also, polydextrose and inulin, as soluble polysaccharide, have been shown to have superior water retention abilities compared to other prebiotics [10], allowing them to retain a large amount of water and increase the viscosity and stability of curd by interacting with milk protein components [4,33].

The greater the stability of the curd, the greater the water-holding capacity of the whey within the gel structure [34]. Centrifugation loss can be used to reflect the change in the water-retaining capacity of yogurt samples [8], and the two are negatively correlated. As shown in Figure 6D, no significant difference (*p* > 0.05) in centrifugation loss (CL) was found among the probiotic yogurt groups, and they were all lower than that of the control samples. However, only the INU-added, XOS-added, and PDX-added yogurt groups showed significantly lower values of CL (*p* < 0.05) than the control group. Inulin and polydextrose are both soluble fibers, and the former is particularly considered a water structuring agent [5]. However, it has been reported that, compared with other polymers such as inulin, polydextrose has small chains and can extend its branched structure more uniformly into casein aggregates [20], resulting in more extensive protein–carbohydrate interactions, better gel stability, and low syneresis [35]. However, inulin has the ability to form microcrystals in milk [5] and exhibits high water retention, in which one inulin molecule binds two water molecules [11]. In addition, inulin can also complex with protein aggregates through H-bridges and form a part of the structural network during fermentation [10] (as shown in Figure 1). Therefore, the higher textural properties in prebiotic yogurt might be partly explained by a better water-retaining ability [10].

## 3. Conclusions

The incorporation of prebiotics affected the rheology, texture, and water-retaining properties of stirred yogurt to varying degrees, depending on the type of prebiotic used. Compared to yogurt without prebiotics, the addition of INU significantly increased the EI and MVI levels of the gel during fermentation, and effectively promoted the development of a more viscous and stiff gel network of yogurt, endowing the product with higher gel stability (higher γ_c_ (*p* > 0.05), G_c_′ and G_c_″ (*p* < 0.05)), which in turn increased the apparent viscosity (η_50_, *p* < 0.05) and texture properties (firmness, consistency, and cohesiveness, *p* < 0.05) of yogurt. Meanwhile, the addition of PDX and XOS exhibited similar functional characteristics, which was reflected in increasing the thixotropy (*p* < 0.05), viscosity (η_50_, *p* < 0.05), and texture properties (*p* < 0.05) of yogurt by improving the viscous (higher MVI level) and elastic (higher EI level) properties of the gel network during fermentation. The enhancement of the texture and rheology of yogurt by these prebiotics was considered to be mainly related to increased water retention (significantly decreased centrifugal loss, *p* < 0.05), which led to enhanced water–milk protein interactions. However, these enhancing effects are less prominent in yogurt supplemented with GOS and FOS. Therefore, these differential effects should be fully considered when selecting prebiotics as functional ingredients in yogurt. This study provides a relatively systematic understanding, to evaluate and compare the effects of several commonly used prebiotics on the structural properties of yogurt. In the future, a larger sample size and wider research scope (including more types of prebiotics and related structural indicators and quality characteristics) will be fully considered, with the aim of establishing a robust and applicable regression model for predicting these characteristics.

## 4. Materials and Methods

### 4.1. Preparation of Stirred Yogurt Samples

UHT milk (3.2% protein, 4.0% fat) (Inner Mongolia Mengniu Dairy (Group) Co., Ltd., Hohhot, China) with a volume of 1.46 L was added to a cylindrical fermenter with a volume of 2.0 L (SY-PA-04-04, Shanghai Shunyi Technology Co., Ltd., Shanghai, China), which was then used as the base to heat up to 45 − 50 °C in the fermentation tank (SY-PA-04-04, Shanghai Shunyi Technology Co., Ltd., Shanghai, China). Subsequently, different prebiotics, including fructooligosaccharide (FOS with a purity of 95% and a degree of polymerization (DP) of 2–8, Chongqing Joywin Natural Products Co., Ltd., Chongqing, China), galactooligosaccharide (GOS with a purity of 85% and a degree of polymerization (DP) of 2–8, Baolingbao Biotechnology Co, Ltd., Shandong, China), inulin (INU with a purity of approximately 100% and an average degree of polymerization (DP) of approximately 23, Chongqing Jiaowang Natural Products Co., Ltd., Chongqing, China), polydextrose (PDX with a purity of ≥90% and an average degree of polymerization (DP) of approximately 12, Chongqing Jiaowang Natural Products Co., Ltd., Chongqing, China), and xylooligosaccharide (XOS with a purity of ≥95% and with a degree of polymerization (DP) of 2–10, Shandong Longlive Bio-Technology Co., Ltd., Shandong, China), were added separately at the same dosage (2.5 g/100 mL) (37.5 g of each prebiotic was weighed and added to 1.46 L of preheated milk) and stirred at 550 rpm using an electronic mechanical stirrer (R30A, FLUKO (Shanghai) Technology Development Co., Ltd., Shanghai, China) until they were completely and evenly mixed with the milk base. Afterwards, the mixture was continuously heated up to 85 °C and kept at that temperature for 20 min, then cooled down to 42 ± 1 °C by circulating water, and fermented by inoculating 0.03% (*w*/*w*) of the mixed starter (*Lactobacillus bulgaricus* (MN-ZLW-003, China General Microbiological Culture Collection Center (CGMCC), CGMCCNO.3818)*: Streptococcus thermophilus* (MN-ZLW-002, China General Microbiological Culture Collection Center (CGMCC), CGMCCNO.3817)) at a ratio of 1:1. When the pH of the fermented milk dropped to 4.50 ± 0.05, the fermentation was terminated. Then, the stirring operation was performed by a mechanical stirrer (R30A, FLUKO (Shanghai) Technology Development Co., Ltd., Shanghai, China) at 550 rpm to disrupt the gel system. The yogurt samples without added prebiotics were used as the control group. The samples of both the prebiotic yogurt group and the control group were kept at 4 °C for 18 h after fermentation for analysis of their textural, rheological, and water-retaining properties.

### 4.2. Microrheological Properties

The gel formation of samples during fermentation was monitored by multi-speckle diffusing wave spectroscopy (MS-DWS) using a Rheolaser Master (Formulaction, Toulouse, France). An inoculated mixture of 20 mL of milk and prebiotics was carefully transferred into a glass tube (inner diameter of 27.5 mm for the Rheolaser Master) to prevent the sample from splashing onto the glass tube wall during operation. Afterwards, the yogurt groups with different added prebiotics were marked with different codes and immediately placed in the Rheolaser Master apparatus, which had been equilibrated to 42 °C in advance. Subsequently, until the pH of each yogurt group successively reached 4.50 ± 0.04 (about 2.0–3.0 h), the fermentation was continued for about 4 h, and then the monitoring was terminated. The collection and analysis of the original data was performed by the software attached to the instrument and directly provided the rheological parameters: EI (elasticity index), MVI (macroscopic viscosity index), and SLB (solid liquid balance).

### 4.3. Rheological Properties

Dynamic rheological measurements including frequency sweep, strain sweep, and apparent viscosity were carried out in a stress-controlled rheometer (Anton Paar MCR302, Austria) using a 1.0 mm gap cone-plate sensor. The yogurt sample was placed on the rheometer’s bottom plate. The sample surface was covered with paraffin oil to prevent evaporation. The top plate was gradually lowered until the predefined gap was 1.0 mm and a cone (50 mm diameter; 1° angle) was applied. The temperature of the plate was set at 25 °C during all measurements.

#### 4.3.1. Frequency Sweep

To ensure that viscoelastic measurements were performed in the linear viscoelastic region (LVR), strain sweep tests were conducted from 0.01% to 50% at a frequency of 1 Hz. Afterwards, samples were subjected to a frequency sweep in the LVR from 0.01 Hz to 10 Hz, with a constant shear strain of 0.5%. The elastic modulus (G’) and viscous modulus (G”) were measured as a function of frequency.

#### 4.3.2. Strain Sweep

The strain sweep range was 0.01–1000% at a fixed angular frequency (ω) of 10 rad/s.

#### 4.3.3. Apparent Viscosity

According to Alina et al. [36], the shear stress at a shear rate of 50 s^−1^ or 100 s^−1^ is a representative parameter for a large deformation test, reflecting the best correlation describing oral perception during the consumption of semi-solid dairy products such as yogurt. In this study, the shear stress at both shear rates was recorded, as the shear rate increased from 0 s^−1^ to 202 s^−1^.

#### 4.3.4. Thixotropy

The thixotropy of the sample was measured at 25 °C using a Rheolab QC rotational shear rheometer equipped with a conical CC 27 probe with a diameter of 20 mm (Rheolab QC, Anton Paar, Austria). The shear rates of the upward and downward curves were 0–100 s^−1^ and 100–0 s^−1^, respectively, and the area enclosed by the two (the so-called thixotropic loop) was used to evaluate the thixotropic behavior of the yogurt.

### 4.4. Textural Properties

The textural attributes of the samples, including firmness, consistency, and cohesiveness, were analyzed by a single compression test with a TA.XT Plus Texture Analyzer equipped with a back extrusion cell disc (A/BE; diameter 40 mm; distance 30 mm). The speed before, during, and after the test was 1.00 mm/s, and the trigger force was Auto-10.0 g.

### 4.5. Water-Retaining Properties

The water-retaining capacity of the yogurt samples was evaluated by the degree of weight loss of yogurt after centrifugation. Briefly, approximately 20 g of yogurt (W_0_) was placed in a 50 mL centrifuge tube, and the total weight (W_1_) of yogurt and the centrifuge tube was obtained. Afterwards, the yogurt was centrifuged at 4500 g for 10 min at 4 °C (Centrifuge 5810 R, Eppendorf AG, Hamburg, Germany) and the expressible moisture was removed, and the total weight (W_2_) of the yogurt and centrifuge tube was obtained again. The centrifugal loss is calculated using the following equation:

Centrifugal loss (%) = (W_1_ − W_2_)/W_0_ × 100% (1)

### 4.6. Statistical Analysis

The statistical analysis of all data was conducted using IBM SPSS Statistical 26.0 software (SPSS Inc., Chicago, IL, USA) and GraphPad Prism 8 software (GraphPad Software Inc., San Diego, CA, Chile). Three independent replicate experiments were performed for all analyzed physico-chemical indicators, and the results were expressed as the mean ± SD. Data normality was determined using the Shapiro–Wilk’s test, and the homogeneity of variance was analyzed using the Levene’s test. For the data that were normally distributed, a one-way analysis of variance (ANOVA) followed by Fisher’s LSD test (for homoscedasticity) or a Dunnett’s T3 test was used to calculate the means with 95% confidence. For non-normally distributed data, a non-parametric Kruskal–Wallis 1-way ANOVA, followed by a Dunn test for multiple comparisons, was applied. A *p* ≤ 0.05 represented a significant difference.

## Figures and Tables

**Table 1:** Critical strain (γ_c_), corresponding critical storage modulus (Gc′), and critical loss modulus (Gc″) of control group and prebiotic yogurt.

Groups	Critical Strain γ_c_ (%)	G_c_′ (Pa)	G_c_″ (Pa)
NT	7.75 ± 0.53 ^ab^	18.68 ± 1.55 ^b^	5.36 ± 0.33 ^b^
GOS	7.40 ± 0.54 ^b^	23.65 ± 1.60 ^a^	6.89 ± 0.68 ^a^
FOS	7.31 ± 0.30 ^b^	24.57 ± 2.03 ^a^	7.02 ± 0.75 ^a^
XOS	8.22 ± 0.34 ^a^	23.14 ± 2.88 ^a^	6.49 ± 0.81 ^a^
INU	8.35 ± 0.34 ^a^	24.55 ± 2.28 ^a^	7.04 ± 0.56 ^a^
PDX	8.21 ± 0.10 ^a^	22.06 ± 0.43 ^ab^	6.33 ± 0.26 ^ab^

Different letters in the same column represent significant differences (*p* < 0.05). NT = control, GOS = galactooligosaccharide, FOS = fructooligosaccharide, XOS = xylooligosaccharide, INU = inulin, PDX = polydextrose.

## Data Availability

All data and materials are available on request from the corresponding author. The data are not publicly available due to ongoing research using a part of the data.
